# N-acetyl Cysteine Overdose Induced Acute Toxicity and Hepatic Microvesicular Steatosis by Disrupting GSH and Interfering Lipid Metabolisms in Normal Mice

**DOI:** 10.3390/antiox13070832

**Published:** 2024-07-11

**Authors:** Ming-Shiun Tsai, Gunn-Guang Liou, Jiunn-Wang Liao, Pin-Yen Lai, Di-Jie Yang, Szu-Hua Wu, Sue-Hong Wang

**Affiliations:** 1 Department of Medicinal Botanicals and Health Applications, Da-Yeh University, Changhua 515006, Taiwan; tsaims1@mail.dyu.edu.tw; 2 Office of Research and Development, College of Medicine, National Taiwan University, Taipei 106319, Taiwan; bogun@gate.sinica.edu.tw; 3 Graduate Institute of Veterinary Pathobiology, National Chung Hsing University, Taichung 402202, Taiwan; jwliao@dragon.nchu.edu.tw; 4 Department of Biomedical Sciences, Chung Shan Medical University, Taichung 402201, Taiwan; r12441005@ntu.edu.tw (P.-Y.L.); s0917045@gm.csmu.edu.tw (D.-J.Y.); r11b43012@ntu.edu.tw (S.-H.W.); 5 Department of Medical Research, Chung Shan Medical University Hospital, Taichung 402201, Taiwan

**Keywords:** NAC overdose, glutathione, microvesicular steatosis, β-oxidation, non-alcoholic fatty liver

## Abstract

N-acetyl cysteine (NAC) is a versatile drug used in various conditions, but the limitations and toxicities are not clear. The acute toxicity and toxicological mechanisms of an intraperitoneal injection of NAC in normal mice were deciphered. The LD50 for male and female BALB/cByJNarl mice were 800 mg/kg and 933 mg/kg. The toxicological mechanisms of 800 mg/kg NAC (N800) were investigated. The serum biomarkers of hepatic and renal indices dramatically increased, followed by hepatic microvesicular steatosis, renal tubular injury and necrosis, and splenic red pulp atrophy and loss. Thus, N800 resulted in mouse mortality mainly due to acute liver, kidney, and spleen damages. The safe dose (275 mg/kg) of NAC (N275) increased hepatic antioxidant capacity by increasing glutathione levels and catalase activity. N275 elevated the hepatic gene expressions of lipid transporter, lipid synthesis, β-oxidation, and ketogenesis, suggesting a balance between lipid production and consumption, and finally, increased ATP production. In contrast, N800 increased hepatic oxidative stress by decreasing glutathione levels through suppressing Gclc, and reducing catalase activity. N800 decreased the hepatic gene expressions of lipid transporter, lipid synthesis, and interferred β-oxidation, leading to lipid accumulation and increasing Cyp2E1 expression, and finally, decreased ATP production. Therefore, NAC doses are limited for normal individuals, especially via intraperitoneal injection or similar means.

## 1. Introduction

N-acetyl cysteine (NAC) is a versatile drug that has been used to treat various conditions. It is the main antidote for acetaminophen (APAP) overdose, which can cause liver failure [1]. It can also improve liver health in patients with non-alcoholic fatty liver disease (NAFLD) or non-alcoholic steatohepatitis (NASH), especially when combined with metformin (MTF) [2,3,4]. Furthermore, NAC has mucolytic effects and, as such, can alleviate respiratory problems, including those related to COVID-19 [5,6]. Research has also indicated that NAC shows analgesic effects [7] and is beneficial in the treatments of neurodegenerative psychiatric diseases [8,9]. NAC is combined with lycopene to prevent damage caused by cisplatin [10]. In some countries, NAC is administered as a dietary supplement [11].

NAC increases intracellular cysteine to promote the synthesis of glutathione (GSH). As it possesses a thiol group, NAC can directly react with reactive oxygen and nitrogen to alleviate oxidative stress or GSH deficiency [12]. The generated GSH by NAC is used to neutralize the N-acetyl-p-benzoquinone imine toxin produced during APAP metabolism [13]. In animal models, NAC has been found to attenuate the inflammatory responses induced by cerebral ischemia or lipopolysaccharides [14,15]. However, according to the meta analyses, an intravenous injection (IV) of NAC to treat sepsis or systemic inflammation does not reduce mortality or complications. On the contrary, giving NAC after 24 h causes cardiovascular instability [16]. Therefore, the effectiveness and mechanism(s) of NAC, when used clinically to treat inflammatory diseases, need to be further explored.

Clinically, NAC is administered orally or intravenously. Since an IV of NAC achieves the desired dose faster than the oral administration of NAC, an IV is usually preferred to treat APAP-poisoning and non-APAP-related acute liver failure for improving the patient’s condition and shortening hospitalization time [17]. The oral administration of NAC is associated with mild side effects, such as nausea, vomiting, itching, and erythema, while an IV of NAC can cause serious reactions, especially at the beginning of infusion when blood NAC level is high. These reactions are allergy-like, with skin symptoms, such as flushing, itching, and angioedema, and systemic symptoms, such as bronchospasm and hypotension [18,19].

The toxicity of NAC varies depending on the route of administration. The LD50 values for oral administration and an intraperitoneal injection (IP) of NAC in rats are 6000 mg/kg and 1205 mg/kg, respectively [20,21]. This result indicates the substantially higher acute toxicity of NAC by IP than oral administration. Our previous results show that an IP of overdose NAC increases mortality and induces system inflammation in both APAP-poisoned mice and normal mice [22]. Otherwise, an IV of overdose NAC causes deaths with renal and brain injuries in the APAP-poisoned patient [23]. However, the dosage effects of NAC by IV lack clinical evidence.

In rodent studies, IP is often favored over IV for its procedural simplicity and accuracy, while maintaining comparable therapeutic efficacy [24]. In this study, the acute toxicities associated with diverse doses of NAC were examined by IP in both male and female inbred mice. Gender-specific variations in NAC sensitivity were investigated, along with an exploration of the potential causes of mortality and hepatotoxic mechanisms induced by high-dose NAC. The molecular mechanisms underlying the NASH development have been explored.

## 2. Materials and Methods

### 2.1. Chemicals and Reagents

NAC, reduced GSH, glutathione peroxidase (GPx), and Tris hydrochloride were obtained from Sigma-Aldrich (St. Louis, MO, USA). Hydrogen peroxide (H_2_O_2_) was purchased from Merck (Darmstadt, Germany). Reaction kits for analyses of malondialdehyde (MDA), triglyceride (TG), catalase (CAT), and GSH were purchased from Elabscience Biotechnology Inc. (Houston, TX, USA). An ATP reaction kit was purchased from Gentex Co. (St. Zeeland, MI, USA). A free fatty acid (FFA) detection kit was obtained from Asia Bioscience Co., Ltd. (Taipei City, Taiwan). Primary antibodies to glutamate cysteine ligase catalytic subunit (Gclc) (ab53179), Forkhead box protein A1 (Foxa1) (ab170933) and cytochrome P450s 2E1 (Cyp2e1) (ab28146) were supplied by Asia Bioscience Co., Ltd. Primary antibodies to glutamate cysteine ligase modifier subunit (Gclm) (GTX114075), peroxisome proliferator-activated receptor alpha (Pparα) (GTX101098), apolipoprotein B (ApoB) (GTX135994), and β-actin (GTX109639) were purchased from Gentex Co. Goat anti-rabbit conjugated horseradish peroxidase antibodies were purchased from Thermo Fisher Scientific (Waltham, MA, USA) and goat anti-mouse conjugated HRP antibodies were purchased from Jackson ImmunoResearch Inc. (West Grove, PA, USA). IncECL solution was supplied by Biokit Biotechnology Inc. (Toufen City, Miaoli County, Taiwan).

### 2.2. Animals and Experimental Designs

Six-week-old male and 7-week-old female BALB/cByJNarl (BALB/c) inbred mice were obtained from the National Laboratory Animal Center (Nangang District, Taipei City, Taiwan). They had free access to sterile water and rodent diet 5001, and were housed in a specific pathogen-free environment at the laboratory animal center of Chung Shan Medical University. The experimental designs followed the animal guidelines of IACUC in the university, with approved protocol number 2454. After allowing the BALB/c mice to adapt for at least one week, they were fasted and weighed. For the acute toxicity assay, male and female mice were randomly divided into six groups (n = 5) based on their body weights and then were intraperitoneally injected with phosphate-buffered saline (PBS) or 600, 800, 900, 1000, and 1200 mg/kg NAC, respectively. The status of these mice was checked every 2 h on the first day and every 4 h until day 7, and then the surviving mice were sacrificed at the end of day 14. For the NAC toxicity study, mice were randomly divided into three groups by their body weights: the control (C), N275 (275 mg/kg NAC), and N800 (800 mg/kg NAC) groups. Mice were anesthetized with isoflurane before blood and tissue collections at 24 h, 48 h, and 96 h. Cardiac puncture was used for obtaining blood samples and hematoxylin and eosin (H&E) staining was used for the histopathological analyses of liver and other major organs. The right liver lobes were stored at −80 °C for biochemical and Western blot analyses.

### 2.3. Serum Biochemical Analyses

Sera were collected from blood samples after centrifugation (3000× *g*, 10 min, 4 °C) and stored at −20 °C until detection. In different NAC treatment groups, serum was collected from each surviving mouse and used to analyze liver (24 h, 48 h, 96 h) and kidney injury indices (24 h, 48 h), and lipid accumulation (48 h, 96 h) (n ≥ 3). In the NAC toxicity study, serum was collected from each surviving mouse at 14 days after IP of ≥800 mg/kg NAC (n = 5). Serum biochemical analyses, including aspartate aminotransferase (AST), alanine aminotransferase (ALT), alkaline phosphatase (ALP), total bilirubin (T-BIL), total proteins (TP), albumin, glucose, blood urea nitrogen (BUN), creatinine (CREA), uric acid (UA), TG, total cholesterol (T-CHO), high-density lipoproteins (HDL), low-density lipoproteins (LDL), calcium (Ca), magnesium (Mg), and phosphorus (P), were measured using Hitachi 7080 blood biochemistry analyzers (Hitachi Ltd., Taipei, Taiwan) and related reagents at the National Laboratory Animal Center [25].

### 2.4. H&E Staining of Multiple Tissue Sections

Tissues were collected for histopathological analyses (24 h, 48 h, 96 h) (n ≥ 3). The right lobe of the liver, kidney, spleen, and other major organs were fixed in 10% neutral buffered formalin and embedded in paraffin. Three-micrometer-thick sections were deparaffinized and rehydrated before stained with H&E. A veterinary pathologist performed liver, kidney, and spleen histological scoring. Lesions were scored from 0 to 5 depending on severity: 0 = none (0%); 1 = minimal (<1%); 2 = slight (1–25%); 3 = moderate (26–50%); 4 = moderate/severe (51–75%); 5 = severe/high (76–100%).

### 2.5. Measurements of Hepatic TG and FFA

Liver tissues (10% *w*/*v*) (48 h, 96 h) (n ≥ 3) were homogenized with isopropanol and chloroform containing 1% Triton X-100, then centrifuged at 10,000× *g* (4 °C) for 10 min to collect supernatants and organic phases for TG and FFA measurements, respectively. TG and FFA were measured using TG (E-BC-K261-M) and FFA (K612-100) assay kits, respectively.

### 2.6. Measurements of Hepatic GSH and CAT Levels

Liver proteins were isolated by homogenization in PBS, and then centrifugation. Protein concentrations were determined by protein assay reagent (Bio-Rad, Hercules, CA, USA). GSH levels (24 h, 48 h, 96 h) (n ≥ 3) and CAT activities (48 h, 96 h) (n ≥ 3) were assayed using GSH (E-BC-K030-M) and CAT (E-BC-K031-S) kits, as previously described [26,27].

### 2.7. Measurements of Hepatic MDA and ATP Levels

Liver proteins were extracted with potassium chloride in PBS containing butylated hydroxytoluene by sonication, and then centrifuged. MDA levels (48 h, 96 h) (n ≥ 3) were measured in the supernatants using an MDA reaction kit (E-BC-K025-M) [28]. Liver tissues were homogenized with ATP buffer and centrifuged. ATP levels (48 h, 96 h) (n ≥ 3) were measured in the supernatants using an ATP reaction kit (GTX85579).

### 2.8. Measurements of Hepatic mRNA Levels of Lipid Metabolism Genes by Real-Time PCR

Liver tissues were collected for the expressions of genes related to lipid metabolism (24 h, 48 h) (n ≥ 3). Using a Trizol reagent and Superscript III system (Thermo Fisher Scientific), liver mRNAs were extracted and reverse transcribed. Then, the mRNA levels of sterol regulatory element-binding protein 1c (Srebp1c), carbohydrate-responsive element-binding protein (Chrebp), Fas, carnitine palmitoyl transferase I (Cpt1), Pparα, peroxisome proliferator-activated receptor-γ coactivator 1-α (Pgc1α), carnitine O-octanoyl transferase (Crot), 3-hydroxy-3-methylglutaryl-CoA synthase 2 (Hmgcs2), acyl-CoA oxidase 1 (Acox1), Foxa1, fatty acid transport protein 2 (Fatp2), Pparγ, and Cyp2e1 were measured by real-time PCR with Fast SYBR Green Master Mix (Thermo Fisher Scientific) on a Bio-Rad CFX system. The used primers are listed in Supplementary Table S1. With 18S rRNA as the internal control, 2(−ΔΔCt) method and 18S rRNA normalization were used to calculate relative gene expression ratios. Ratios in the C group were all defined as 1.0.

### 2.9. Western Blotting

Western blotting was performed as previously described [29]. Liver tissues (24 h, 48 h, 96 h) (n ≥ 3) were homogenized in a radio-immunoprecipitation assay buffer, and then centrifuged (10,000× *g*, 4 °C, 20 min). After the detection of protein concentrations, forty-microgram proteins in each sample were separated by 12% sodium dodecyl sulfate-polyacrylamide gel electrophoresis, then transferred to polyvinylidene difluoride membrane (Merck Millipore, Boston, MA, USA). Membranes were blocked with 5% bovine serum albumin in Tris buffered saline plus Tween 20 (TBST) at room temperature for 1 h or at 4 °C overnight, followed by incubation with primary antibodies against Gclc (1:3000), Gclm (1:3000), Pparα (1:3000), Foxa1 (1:3000), ApoB (1:3000), Cyp2e1 (1:1000), or β-Actin (1:1000) at 4 °C overnight. Membranes were washed with TBST, followed by incubation with secondary antibodies. The signals of β-Actin were used as the loading control. Protein signals were detected with IncECL solution and carried out quantification with AlphaEaseFC 6.0 software.

### 2.10. Statistical Analysis

At least three independent experiments were performed for all experiments. Data were expressed as mean ± standard deviation (SD) using Microsoft Office Excel 2016 software (Microsoft Crop., Redmond, WA, USA). Statistical analyses were performed using SPSS for Windows, version 25 (SPSS, Inc., Chicago, IL, USA) and one-way ANOVA, followed by Dunnett’s test for comparisons of data between the N275 or N800 group and the C group, and between the N275 and N800 groups. A value of *p* < 0.05 indicated statistical significance.

## 3. Results

### 3.1. LD50 of Male and Female Mice following IP of NAC

**Figure 1:**
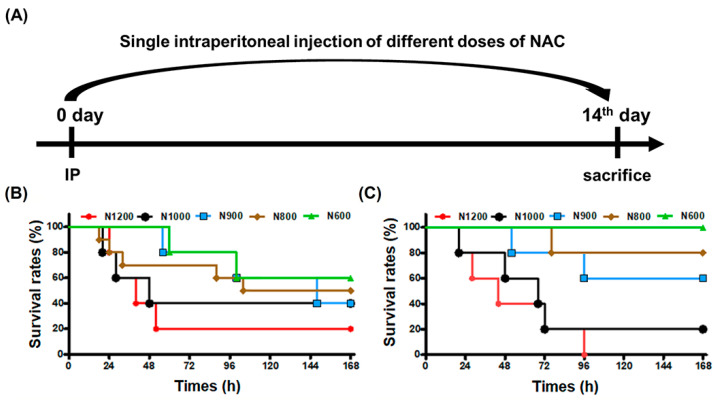
The flowchart (**A**) and the curves of the survival rates of mice (**B**,**C**) were shown for the acute toxicity assay after a single IP of different doses of NAC. The curves of the survival rates of male (**B**) and female (**C**) mice for an IP of indicated doses of NAC were shown in different colors.

The acute toxic effects of different NAC doses administered via single IP in normal mice were assayed for the survival of male and female mice over a 14-day period (Figure 1A). The survival rates of mice were observed for 7 days (Figure 1B,C). NAC caused mortalities in both genders, mostly within 18–96 h. These survival results suggest that mice can recover from NAC toxicity after 96 h. From N600 (600 mg/kg NAC) to N1200 (1200 mg/kg NAC), the survival rates of male mice dropped from 60% to 20% (Figure 1B) and those of female mice dropped from 100% to 0% (Figure 1C). The LD50 values of NAC for males and females were 800 mg/kg and 933 mg/kg, respectively, showing that males are more vulnerable than females to an IP of NAC.

### 3.2. Histopathological Findings and Serum Biochemical Values of Surviving Male and Female Mice after High-Dose NAC Treatment

**Figure 2:**
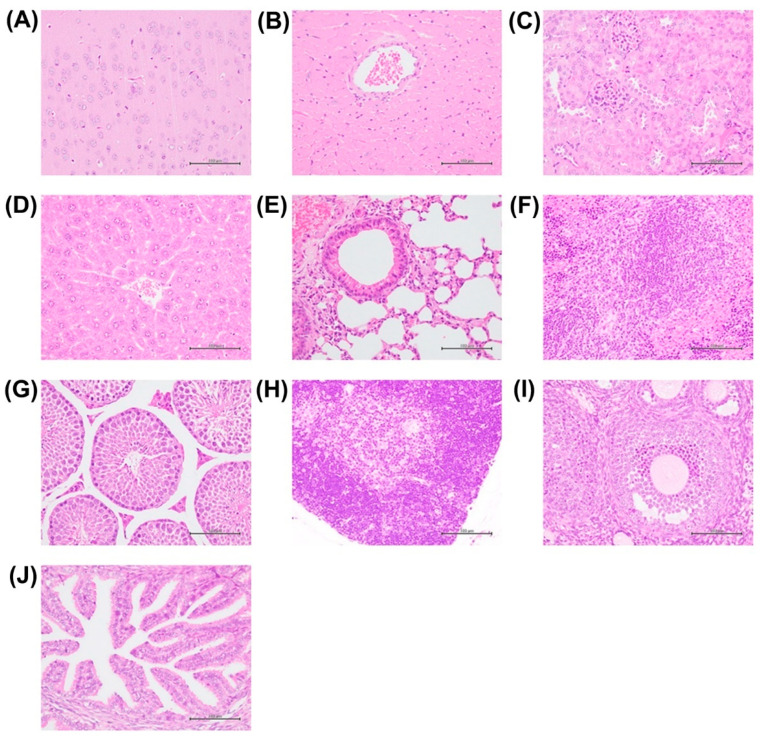
Histopathological findings of the major organs of the chosen surviving male mouse (**A**–**H**) and female mouse (**I**,**J**) after an IP of high-dose NAC at the end of day 14. No significant change in the brain (**A**), heart (**B**), kidney (**C**), liver (**D**), lung (**E**), spleen (**F**), testis (**G**) or thymus (**H**) in the male mouse, or ovary (**I**) or oviduct (**J**) in the female mouse were found. Scale bars: 100 μm.

We examined the main organs (Figure 2) of male and female mice that survived for 14 days after a single IP of high-dose (≥800 mg/kg) NAC. No major tissue or cell damage was seen in the brain, heart, kidney, liver, lung, spleen, or testis and thymus of the chosen male mouse or ovary and oviduct of the chosen female mouse on histopathological analyses. These results indicate that the major organs of the chosen surviving mice were not severely damaged or could be repaired within 14 days after NAC-induced injury. They also suggested that a single IP of NAC induced acute toxicity.

We then analyzed the blood biochemistry of mice that had survived for 14 days after a single IP of high-dose NAC (≥800 mg/kg, Table 1). Among males, the liver function indices of ALP, T-BIL, and ALB were significantly lower in the NAC group than in the C group (ALP: *p* < 0.001; T-BIL: *p* < 0.05; ALB: *p* < 0.01). Among females, T-BIL, TP, and ALB were significantly lower in the NAC group than in the C group (T-BIL: *p* < 0.01; TP: *p* < 0.001; ALB: *p* < 0.01). ALP was also lower in the NAC group than in the C group, but with no significant difference. The low TP and ALB values in the NAC group suggest chronic liver and/or kidney diseases in surviving mice [30,31], despite normal-looking liver and kidney tissues (Figure 2).

In terms of kidney function indices, BUN and CREA were significantly lower but UA was significantly higher in the NAC group than in the C group in males (all *p* < 0.05). In the NAC female group, BUN was lower but UA was higher than the C group but no significance. Elevations in BUN and CREA are associated with renal dysfunction and decreases with cirrhosis or renal injury. UA, a purine metabolite, also increased in line with kidney failure or inflammation [32,33]. The elevated UA but reduced BUN in the NAC group suggests renal injury or inflammation and is more severe in males than in females (Table 1).

Serum glucose was increased, but not significantly, in both NAC males and females (Table 1). For serum lipids, NAC treatment lowered TG, T-CHO, and HDL in both genders (male: *p* < 0.01 for TG and T-CHO, *p* < 0.05 for HDL; female: *p* < 0.001 for all) (Table 1). NAC treatment also decreased LDL in males (*p* < 0.001), but was not significant in females. The T-CHO/HDL and LDL/HDL ratios, risk indicators of lipid metabolism, were both lower in the NAC male mice than in the control male mice, but only the LDL/HDL ratio was significant (*p* < 0.05). However, these ratios were higher in NAC-treated females than in control females, but with no significance. These serum lipid results indicated that the lipid metabolisms are affected in both male and female mice treated with high-doses NAC.

The serum Ca levels in both NAC-treated males and females were decreased, but only females showed a significant decrease compared with the control females (*p* < 0.001) (Table 1). Both serum Mg and P levels were also decreased in NAC-treated males. In contrast, serum Mg and P levels were increased in NAC-treated females, but only the P levels showed a significant decrease compared with the control females (*p* < 0.01). This phenomenon suggests a possible association with hypoparathyroidism or chronic renal dysfunction, especially in high-dose NAC treated females [34].

We found that an IP of high-dose NAC in normal mice could be lethal or cause liver and kidney damages over a 14-day period. High-dose NAC reduced blood lipids and affected lipid metabolisms in both genders. The body weights of mice that received high-dose NAC and then died were obviously decreased. Since males showed a higher mortality rate than females (Figure 1), the weight changes were −2.28 ± 1.20 for males and −1.48 ± 0.72 for females. The body weight losses were obviously reduced for the surviving mice that received high-dose NAC. Male and female mice survived for 14 days after an IP of high-dose NAC showed body weight changes of 1.44 ± 0.84 for males and −0.06 ± 0.80 for females. However, these body weight changes were significantly lower than the control mice that received the same volume of PBS (males: 4.43 ± 0.37, *p* < 0.001; females: 1.81 ± 0.91, *p* < 0.01).

### 3.3. Toxic Mechanisms of NAC Determined by Hepatic and Renal Biochemical Indices in Sera

**Figure 3:**
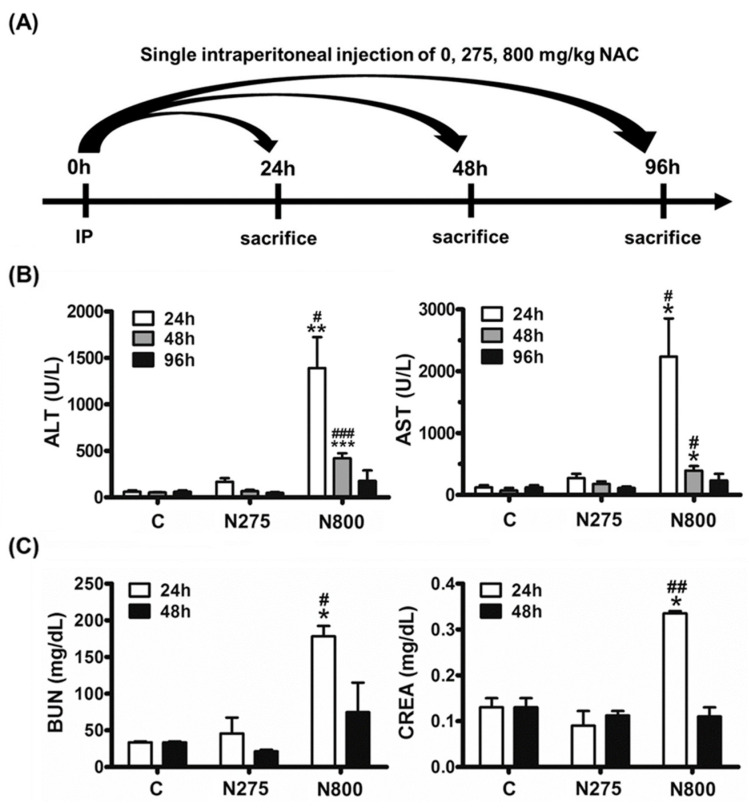
The flowchart (**A**) and serum liver and kidney biochemical indices in normal male mice (**B**) after an IP of different doses of NAC. Serum ALT and AST activities (**B**), and serum BUN and CREA levels (**C**) of mice in the indicated groups were sacrificed at the indicated time points. * (*p* < 0.05), ** (*p* < 0.01), and *** (*p* < 0.001) denote significant differences compared with the C group; # (*p* < 0.05), ## (*p* < 0.01), and ### (*p* < 0.001) denote significant differences compared with the N275 group.

According to our previous article, the IPs of NAC with 275 mg/kg and 800 mg/kg are safe and toxic, respectively, in both APAP-poisoned and normal mice [22]. Since males are more sensitive than females for the IPs of NAC, male mice were used for the NAC toxicity study. In this study, normal male mice were administered varying doses of NAC: 0 mg/kg (C group, PBS control); 275 mg/kg (N275, an optimal dose for our previous APAP-poisoned study); and 800 mg/kg (N800, LD50 for male mice in this study). They were euthanized at 24 h, 48 h, or 96 h post-NAC administration (Figure 3A) and various tests were conducted to identify the possible cause(s) of death in mice of the N800 group.

N800 group mice had significantly higher serum ALT and AST activities than C or N275 group mice at 24 h and 48 h (all *p* < 0.05) (Figure 3B), indicating acute liver injury in N800 group mice. At 96 h, serum ALT/AST mean values in N800 group mice were still higher than those in the C and N275 groups, but the differences were not significant. The declines of the serum hepatic biochemical indices indicated that almost all hepatocytes that can be damaged by N800 have been damaged within 24 h, and some hepatocytes in the surviving mice have repaired or regenerated after injury within 48–96 h. Thus, there are few hepatocytes that can be continually damaged by N800 from 48 h to 96 h, so the serum liver biochemical indices have declined from 24 h to 96 h. Similarly, N800 group mice had significantly higher serum BUN/CREA levels than C or N275 group mice at 24 h (all *p* < 0.05), indicating acute kidney injury in N800 group mice. At 48 h, the serum BUN mean value in N800 group mice was still higher than in C or N275 group mice, but the differences were not significant (Figure 3C). The results imply that the kidneys of the surviving mice have largely healed at 48 h. In conclusion, the serum liver and kidney biochemical indices confirmed that N800 treatment induced acute liver and kidney toxicities in normal male mice, as did the blood biochemistry results (Table 1).

### 3.4. Toxic Mechanisms of NAC Determined by Major Organ Analyses

**Figure 4:**
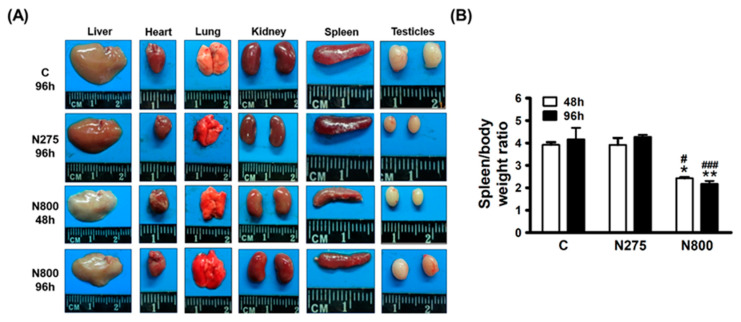
Appearance of major organs (**A**) and the ratios of spleen weight to body weight (**B**) of normal male mice after an IP of indicated doses of NAC at indicated time points. The ratios of spleen weight to body weight of mice in the indicated groups sacrificed at indicated time points were calculated (**B**). * (*p* < 0.05) and ** (*p* < 0.01) denote significant differences compared with the C group; # (*p* < 0.05) and ### (*p* < 0.001) denote significant differences compared with the N275 group.

To elucidate the potential toxic mechanism(s) in normal male mice, we examined the major organs at 48 h and 96 h after an IP of N800. We found no significant changes in organ weights, except for the spleen, liver, and testes (Figure 4A). In the N800 group, white liver lobes and shrunken spleen were noted when compared with C or N275 groups. Furthermore, both N800 and N275 group mice had smaller testes than C group mice. The ratios of spleen weight to body weight in N800 group were much lower than in the other groups (Figure 4B, all *p* < 0.05).

### 3.5. Toxic Mechanisms of NAC Determined by Hepatic, Renal, and Splenic Histopathology

**Figure 5:**
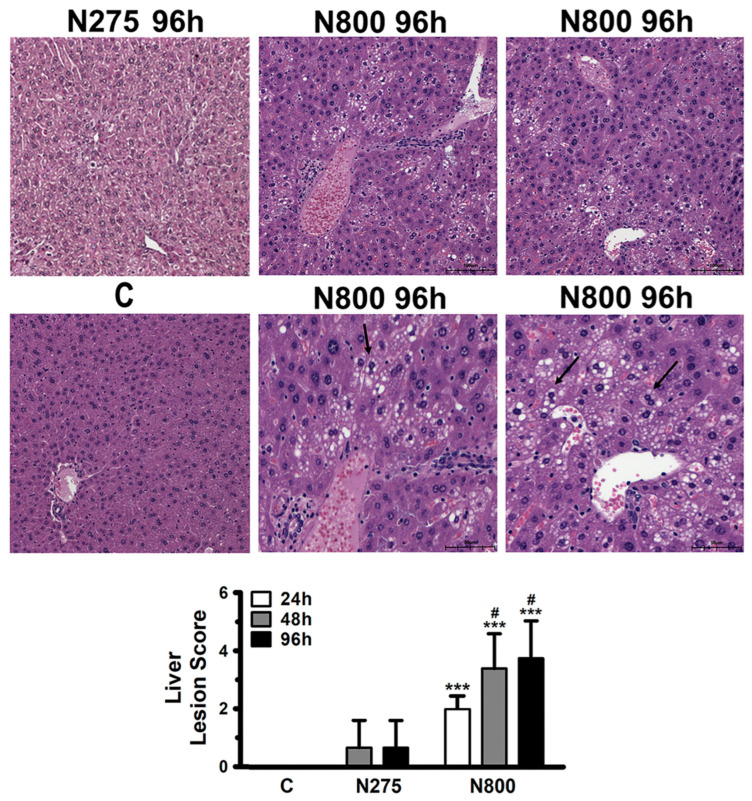
Histopathological findings of liver sections and liver lesion scores in normal male mice after an IP of indicated doses of NAC. Normal hepatocytes were found in the C group (middle-left panel). Multifocal slight fatty changes (arrows) in the portal area were noted in the N800 group at 96 h (middle-center and -right panels). Liver lesion scores were calculated for the indicated groups at the indicated time points (lower panel). *** (*p* < 0.001) denotes significant difference compared with the C group; # (*p* < 0.05) denotes significant difference compared with the N275 group. Scale bar: 100 μm (upper panels and the C group) and 50 μm (middle panels).

The results of serum liver and kidney indices, organ weights, and morphology assessments suggest that an IP of N800 induces acute hepatotoxicity, nephrotoxicity, and splenotoxicity in normal male mice, resulting in mortality. Histopathological analyses were conducted to investigate the pathological changes in the liver, kidney, and spleen of male mice across different time points and groups (Figure 5). Liver sections stained with H&E revealed more hepatocellular damages in the N800 group, based on fatty change, microvesicular, portal area, and multifocal criteria, than in the N275 group (both *p* < 0.05) or C group (all *p* < 0.001). Hepatocytes in the portal area of the N800 group had small lipid vacuoles at 96 h (Figure 5, middle-center and -right panels), suggesting small droplet accumulations in the liver cells. These findings, along with the elevated serum ALT/AST activities (Figure 3B), indicated that N800 caused acute liver injury and microvesicular steatosis (MS) formation. Otherwise, the serum liver biochemical indices have declined from 24 h to 96 h (Figure 3B). However, the repair or regeneration of hepatocytes takes a long time, so the liver lesion scores have continually increased within 48 h to 96 h (Figure 5).

**Figure 6:**
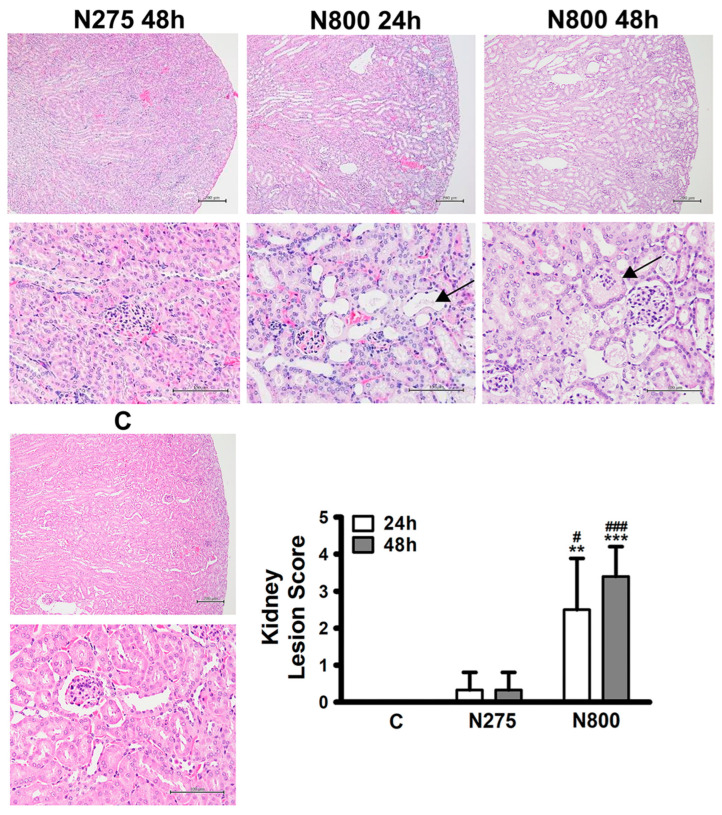
Histopathological findings of kidney sections and kidney lesion scores in normal male mice after an IP of indicated doses of NAC. Multifocal, tubular degeneration/necrosis with dilation and cellular debris (arrows) in the cortex of kidneys were noted in the N800 group at 24 h and 48 h (middle-center and -right panels). Kidney lesion scores were calculated for the indicated groups at the indicated time points (lower right panel). ** (*p* < 0.01) and *** (*p* < 0.001) denote significant differences compared with the C group; # (*p* < 0.05) and ### (*p* < 0.001) denote significant differences compared with the N275 group. Scale bar: 200 μm (upper panel of each section) and 100 μm (lower panel of each section).

Since the kidney biochemical indices, BUN and CREA, in the N800 group had declined to levels similar to the C or the N275 group at 48 h (Figure 3C), histopathological analyses of kidney sections and kidney lesion scores were performed at 24 h and 48 h. The H&E staining of kidney sections showed tubular degeneration, necrosis, and dilation in the renal cortex of the N800 group at 24 h and 48 h (Figure 6, upper-center and -right and middle-center and -right panels). The kidney injury scores of each group of mice were statistically analyzed. The N800 group mice had significantly higher kidney injury scores than the C and N275 group mice at 24 h and 48 h (all *p* < 0.05). Since the serum BUN/CREA levels of the N800 group were significantly higher than those of the C and N275 groups at 24 h and 48 h (Figure 3C), the results of kidney histopathology also demonstrated that an IP of N800 induced acute kidney injury.

**Figure 7:**
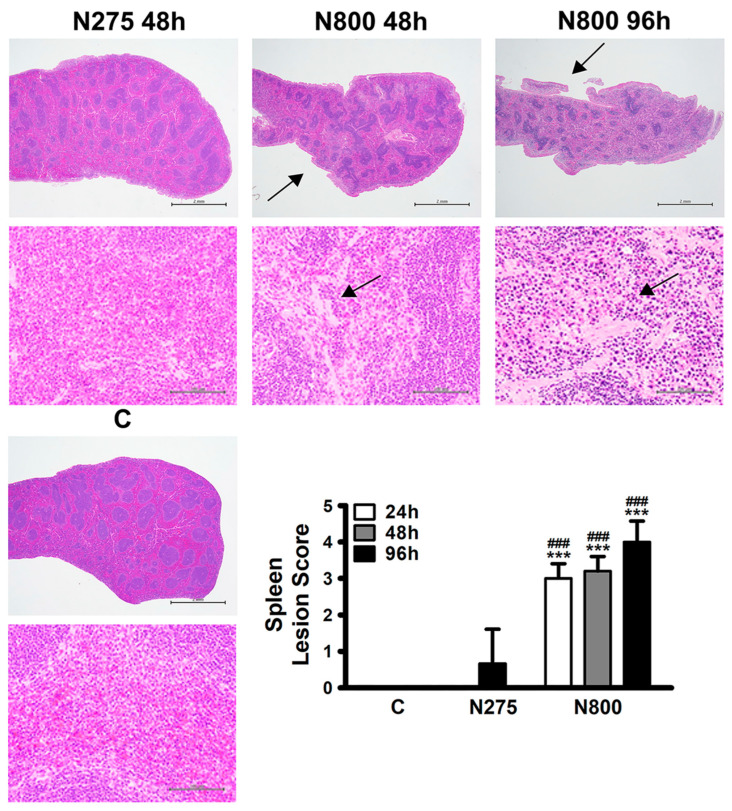
Histopathological findings of spleen sections and spleen lesion scores in normal male mice after an IP of indicated doses of NAC. Normal white and red pulp were found in spleens of both the C and N275 groups at 48 h (meddle- and lower-left panels). Red pulp with a diffuse, a moderate to severe cellular decrease, and contracted trabecula and capsule (arrows) due to the atrophy of the red pulp, were noted in the N800 group at 48 h and 96 h (middle-center and -right panels). Spleen lesion scores were calculated for the indicated groups at the indicated time points (lower-right panel). *** (*p* < 0.001) denotes significant difference compared with the C group; ### (*p* < 0.001) denotes significant difference compared with the N275 group. Scale bar: 2 mm (upper panel of each section) and 100 μm (lower panel of each section).

Since shrunken spleen and low spleen weight to body weight ratios were obtained in the N800 group (Figure 4), histopathological analyses of spleen sections and spleen lesion scores were performed. The H&E staining of spleen sections revealed diffuse and moderate to severe red pulp depletion in the N800 group at 48 h and 96 h post-NAC exposure, along with trabecular and capsular shrinkage due to red pulp atrophy (Figure 7, middle-center and -right panels). The N800 group mice had significantly higher spleen injury scores than the C and N275 group mice at 24 h, 48 h and 96 h (Figure 7, lower-right panel, all *p* < 0.001).

### 3.6. Effects of NAC on Lipid Metabolism

**Figure 8:**
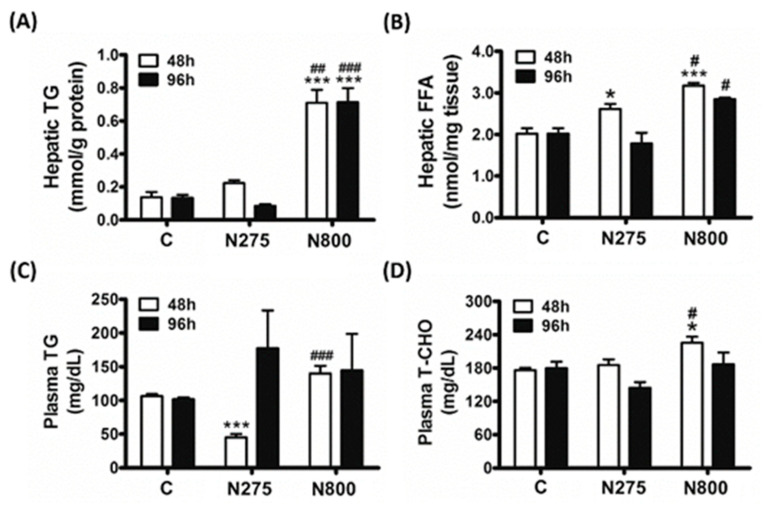
Hepatic TG (**A**) and FFA (**B**) levels and plasma TG (**C**) and T-CHO (**D**) concentrations in normal male mice after an IP of indicated doses of NAC, which were sacrificed at the indicated time points. * (*p* < 0.05) and *** (*p* < 0.001) denote significant differences compared with the C group; # (*p* < 0.05), ## (*p* < 0.01), and ### (*p* < 0.001) denote significant differences compared with the N275 group.

High-dose NAC caused acute cell damages in liver, kidney, and spleen, which may explain mouse mortality. In the liver, the main metabolic organ, high-dose NAC induced MS formation (Figure 5, middle-center and -right panels). We further studied how NAC affects hepatic lipid metabolism by measuring the hepatic TG and FFA levels (Figure 8A,B). TG levels significantly increased in the N800 group at 48 h and 96 h (*p* < 0.001) compared with the C group, and were also higher than those in the N275 group (48 h: *p* < 0.01; 96 h: *p* < 0.001) (Figure 8A). FFA levels significantly increased in the N275 group at 48 h (*p* < 0.05) compared with the C group, but normalized at 96 h (Figure 8B). FFA levels also significantly increased in the N800 group but were only significant at 48 h (*p* < 0.001) compared with the C group, and were higher than in the N275 group at both 48 h and 96 h (both *p* < 0.05). Thus, N275 temporarily increased hepatic FFA accumulation but not TG level. N800 enhanced both hepatic FFA and TG levels, with high TG levels persisting for 96 h.

We also measured the plasma TG and T-CHO levels (Figure 8C,D). N275 showed lower TG levels at 48 h (*p* < 0.001) compared with the C group, but with increased TG levels at 96 h (Figure 8C). However, N275 did not significantly affect plasma T-CHO levels (Figure 8D). N800 significantly elevated TG levels compared with the C group (*p* < 0.001) and T-CHO levels compared with the C and N275 groups (both *p* < 0.05) at 48 h. At 96 h, N800 maintained high TG levels, but T-CHO levels were similar to the C group. Both plasma TG and T-CHO levels in N800 were higher than those in N275 at 48 h (TG: *p* < 0.001; T-CHO: *p* < 0.05) but similar at 96 h.

In brief, N275 briefly increased hepatic FFA levels but decreased plasma TG levels at 48 h, with normalization at 96 h. N800 boosted hepatic TG and FFA levels for 96 h and increased plasma TG and T-CHO levels at 48 h. Thus, the N800-induced hepatic MS formation and liver damage may be due to excess fat accumulation. Since the surviving mice from the IP of high-dose NAC showed significant decreases in serum lipids (Table 1), the decreases in serum lipids at the end of day 14 may result from consumptions of boosted hepatic and plasma lipids (Figure 8).

### 3.7. Effects of NAC on Hepatic GSH and ATP Syntheses

**Figure 9:**
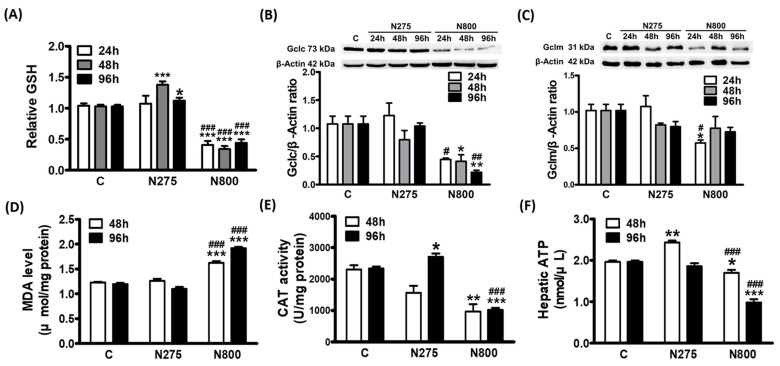
Relative hepatic GSH levels (**A**), Gclc (**B**) and Gclm (**C**) expressions, MDA levels (**D**), CAT activities (**E**) and ATP concentrations (**F**) in normal male mice after an IP of indicated doses of NAC were sacrificed at the indicated time points. * (*p* < 0.05), ** (*p* < 0.01), and *** (*p* < 0.001) denote significant differences compared with the C group; # (*p* < 0.05), ## (*p* < 0.01), and ### (*p* < 0.001) denote significant differences compared with the N275 group.

We investigated the effects of different doses of NAC, a GSH precursor, on hepatic GSH levels and synthetic enzyme levels at the indicated time points. The N275 group had significantly higher GSH levels than the C group at 48 h and 96 h (48 h: *p* < 0.001; 96 h: *p* < 0.05) (Figure 9A). However, the N800 group had significantly lower GSH levels at all these time points than the C and N275 groups (all *p* < 0.001). GSH synthesis depends on glutamate cysteine ligase (Gcl), which is composed of catalytic (Gclc) and modifier (Gclm) subunits. The expression levels of Gclc and Gclm were measured by Western blotting (Figure 9B,C). There were no significant differences in Gclc expressions between the N275 and C groups, with slightly lower Gclm expressions in N275 at 48 h and 96 h. The N800 group showed significantly lower Gclc expressions than the C group at 48 h (*p* < 0.05) and 96 h (*p* < 0.01) and the N275 group at 24 h (*p* < 0.05) and 96 h (*p* < 0.01). In addition, Gclm expressions were also lower in the N800 group than in the C and N275 groups, but these differences were only significant at 24 h (both *p* < 0.05). The results in Figure 9A–C indicate that an IP of N800 reduced GSH synthesis by downregulating Gclc and Gclm, resulting in intensely decreased hepatic GSH levels.

The levels of the lipid peroxidation product, MDA, in the liver, reflect the oxidative stress and GSH consumptions. Figure 9D showed no significant difference in MDA levels between the N275 and C groups at two identified time points. However, the N800 group mice had much higher MDA levels than the C and N275 group mice at both time points (all *p* < 0.001). These results suggest that N800 induces the hepatic TG and FFA accumulations (Figure 8A,B) and depletes hepatic GSH level (Figure 9A), resulting in increases in lipid peroxidation (Figure 9D) and oxidative stress. The hepatic antioxidant enzyme, CAT, and its activity, was also measured (Figure 9E). The CAT activities in the N275 group mice were slightly lower than in the C group mice at 48 h but significantly higher at 96 h (*p* < 0.05). The CAT activities in the N800 group mice were much lower than in the C group mice at both time points (48 h: *p* < 0.01; 96 h: *p* < 0.001), and were slightly lower at 48 h but significantly lower than in the N275 group mice at 96 h (*p* < 0.001). The decrease in hepatic CAT activity in the N800 group mice further increased the oxidative stress from the IP of N800.

Aerobic respiration in mitochondria creates reactive oxygen species (ROS), which are harmful molecules. GSH is a molecule that can neutralize ROS and protect mitochondria. If there is not enough GSH, ROS can damage mitochondria and block ATP production. The results in Figure 9F show that N275 group mice had significantly higher hepatic ATP levels than C group mice at 48 h (*p* < 0.01), but similar at 96 h. However, ATP levels were significantly lower in the N800 group than in the C group (48h: *p* < 0.05; 96h: *p* < 0.001) and in the N275 group (both *p* < 0.001). Thus, ATP levels as well as GSH levels showed the same patterns, though inverted, in both N275 and N800 groups (Figure 9A,F). Since GSH levels were low and MDA levels were high in the N800 group, these results indicate that an IP of N800 in normal mice increases oxidative stress, which affects ATP synthesis in the liver.

### 3.8. Effects of NAC on Hepatic Lipid Metabolism

**Figure 10:**
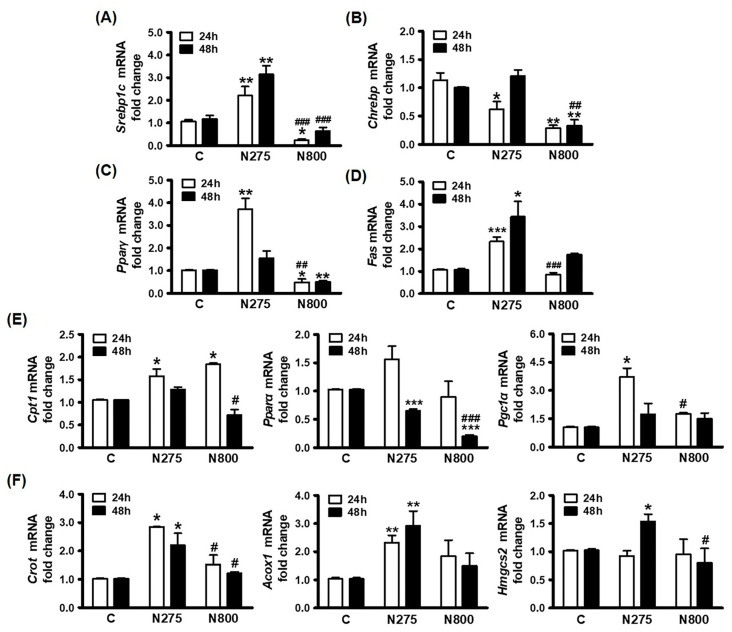
Hepatic lipid metabolic-related gene expressions in normal male mice after an IP of indicated doses of NAC, which were sacrificed at the indicated time points. The expression levels of analyzed genes in the C group were all designated 1.0-fold. The relative expression levels of the analyzed genes in the N275 and N800 groups at indicated time points were calculated. The results shown were fold changes in expressions of (**A**) *Srebp1c*, (**B**) *Chrebp*, (**C**) *Pparγ* and (**D**) *Fas*; (**E**) *Cpt1* (left), *Pparα* (middle), and *Pgc1α* (right); (**F**) *Crot* (left), *Acox1* (middle), and *Hmgcs2* (right). * (*p* < 0.05), ** (*p* < 0.01), and *** (*p* < 0.001) denote significant differences compared with the C group; # (*p* < 0.05), ## (*p* < 0.01), and ### (*p* < 0.001) denote significant differences compared with the N275 group.

Based on the results of this study, a single IP of high-dose NAC in normal mice interfered plasma lipid levels (Table 1, Figure 8C,D), caused hepatic TG and FFA accumulations (Figure 8A,B, Figure 5), and increased hepatic MDA levels (Figure 9D). To investigate the impacts of different NAC doses on catabolic and anabolic pathways of hepatic lipids, we measured gene expression levels involved in the de novo lipogenesis process (Figure 10A–D). The N275 group had significantly increased expressions of *Srebp1c* at 24 h and 48 h (both *p* < 0.01), *Pparγ* at 24 h (*p* < 0.01), and *Fas* 24 h and 48 h (24 h: *p* < 0.001; 48 h: *p* < 0.05), but decreased *Chrebp* at 24 h (*p* < 0.05), restored at 48 h (Figure 10A–D) compared with C group. The N800 group had significantly decreased expressions of *Srebp1c* at 24 h (*p* < 0.05), and *Chrebp* and *Pparγ* at both time points (all *p* < 0.05) compared with the C group. The N800 group also had significantly decreased expressions of *Srebp1c* at both 24 h and 48 h (both *p* < 0.001), *Chrebp* at 48 h (*p* < 0.01)*, Pparγ* at 24 h (*p* < 0.01), and *Fas* at 24 h (*p* < 0.001) compared with the N275 group. In brief, N275 treatment stimulated hepatic de novo lipogenesis by upregulating *Srebp1c*, *Pparγ*, and *Fas*, while N800 treatment inhibited *Srebp1c*, *Pparγ*, and *Chrebp* but maintained de novo lipogenesis.

In addition, we analyzed the effects of NAC on the expressions of mitochondrial lipid β-oxidation related genes. The results in Figure 10E show that the N275 group had transiently upregulated expressions of Cpt1 (*p* < 0.05), *Pparα*, and *Pgc1α* (*p* < 0.05) at 24 h, but downregulated *Pparα* at 48 h (*p* < 0.001) compared with the C group. The N800 group only had a transiently increased *Cpt1* expression at 24 h (*p* < 0.05) but had a significantly reduced *Pparα* expression at 48 h (*p* < 0.001) compared with C group. Compared with N275, N800 also had decreased *Cpt1* (*p* < 0.05) and *Pparα* (*p* < 0.001) expressions at 48 h and *Pgc1α* (*p* < 0.05) expression at 24 h. These results suggest that N275 treatment stimulates mitochondrial lipid β-oxidation by increasing *Cpt1*, *Pparα*, and *Pgc1α* expressions. Despite a transient increase in *Cpt1* expression, N800 treatment suppresses *Pparα* expression and then interferes mitochondrial lipid β-oxidation.

Moreover, we evaluated the gene expressions involved in peroxisomal lipid β-oxidation (Figure 10F, left and middle panels). The N275 group showed significantly higher expressions of *Crot* (both *p* < 0.05) and *Acox1* (both *p* < 0.01) than the C group at both time points. The N800 group exhibited similar *Crot* and *Acox1* mRNA levels to the C group, but a significantly lower *Crot* level than the N275 group at both time points (both *p* < 0.05). We also assessed expressions of Hmgcs2, a key rate-limiting enzyme for ketone body synthesis (Figure 10F, right panel). The N275 group showed significantly higher *Hmgcs2* level at 48 h than the C group (*p* < 0.05). The N800 group exhibited similar levels of *Hmgcs2* expressions at both time points compared to the C group, but had a significantly lower *Hmgcs2* level than the N275 group at 48 h (*p* < 0.05). In brief, these findings indicate that N275 treatment enhances peroxisomal lipid β-oxidation and ketone body synthesis by upregulating *Crot*, *Acox1*, and *Hmgcs2*. Compared with N275 treatment, N800 treatment reduces peroxisomal lipid β-oxidation and ketone body synthesis by downregulating *Crot* and *Hmgcs2*. Therefore, N275 treatment stimulates both the catabolic and anabolic pathways of hepatic lipids, resulting in a balance of lipid metabolism, N800 treatment interferes with mitochondrial lipid β-oxidation and reduces both peroxisomal lipid β-oxidation and ketone body synthesis, leading to hepatic MS.

### 3.9. Effects of NAC on NAFL-Related Gene Expressions: Foxa1, Cyp2e1, and Pparα

**Figure 11:**
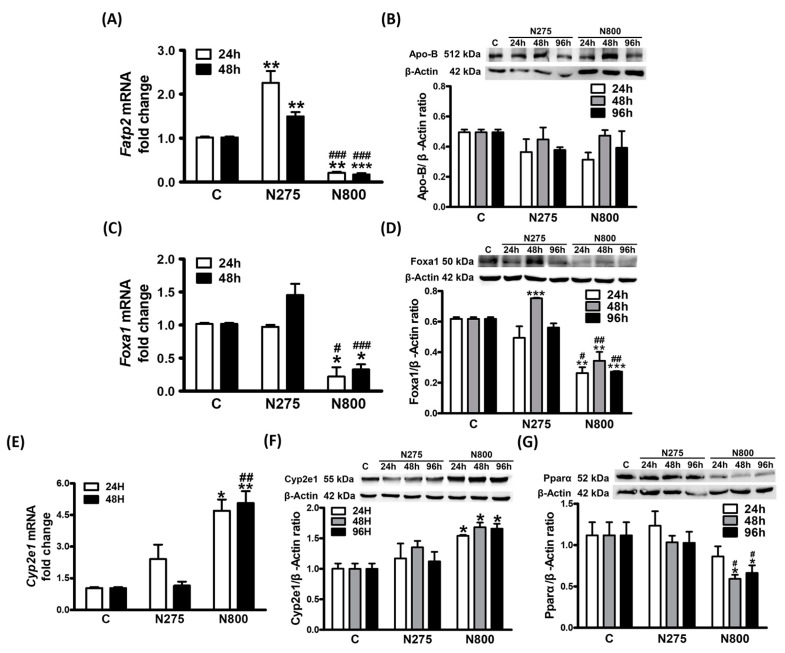
NAFL-related gene expressions in normal male mice after an IP of indicated doses of NAC were sacrificed at the indicated time points. The expression levels of analyzed genes in the C group were all designated 1.0-fold. The relative gene expression levels in the N275 and N800 groups were calculated at indicated time points. Fold changes in gene expressions of *Fatp2* (**A**), *Foxa1* (**C**) and *Cyp2e1* (**E**) were shown. The protein expression ratios of ApoB (**B**), Foxa1 (**D**), Cyp2e1 (**F**), and Pparα (**G**) to β-Actin in the C, N275, and N800 groups were calculated at indicated time points, respectively. * (*p* < 0.05), ** (*p* < 0.01), and *** (*p* < 0.001) denote significant differences compared with the C group; # (*p* < 0.05), ## (*p* < 0.01), and ### (*p* < 0.001) denote significant differences compared with the N275 group.

We also examined the expressions of Fatp2 (Figure 11A), the major transporter of FFA into the liver, and Apo-B (Figure 11B), which is involved in the secretion of TG from the liver. The N275 group demonstrated significantly higher *Fatp2* expressions than the C group at two time points (both *p* < 0.01), while the N800 group demonstrated significantly lower *Fatp2* expressions than the other two groups (all *p* < 0.01). Although Apo-B protein levels were similar in all groups at three time points, the N800 group showed the lowest Apo-B level at 24 h. These results indicate that N275 treatment increases hepatic FFA uptake and slightly decreases TG secretion, so N275 treatment increases hepatic FFA level at 48 h (Figure 8B) and slightly increases hepatic TG level, but significantly decreases plasma TG level at 48 h (Figure 8A,C). Otherwise, N800 treatment decreases hepatic FFA uptake and also slightly decreases TG secretion at 24 h, so N800 treatment significantly increases hepatic TG levels at both 48 h and 96 h (Figure 8A).

While investigating the alterations of transcription factors in drug-induced steatosis, we noted a common pattern of downregulations of Foxa1, Hex, and Srebp1c [35]. Since *Srebp1c* expressions were markedly downregulated in the N800 group at two time points (Figure 10A), we examined whether *Foxa1* expression was also affected. The results in Figure 11C,D revealed concurrent increases in relative *Foxa1* mRNA and Foxa1 protein levels at 48 h in the N275 group compared with the C group, but only the increase in Foxa1 protein level was significant (*p* < 0.001). In the N800 group, *Foxa1* mRNA levels (all *p* < 0.05) and Foxa1 protein levels (24 h and 48 h: *p* < 0.01; 96 h: *p* < 0.001) were persistently and significantly reduced when compared with the C group. Compared with the N275 group, the *Foxa1* mRNA levels (24 h: *p* < 0.05; 48 h: *p* < 0.01) and Foxa1 protein levels (24 h: *p* < 0.05; 48 h and 96 h: *p* < 0.01) of the N800 group were also persistently and significantly reduced. The middle panel of Figure 10E showed that N800 treatment downregulated *Pparα* at 48 h and impaired mitochondrial lipid β-oxidation. Pparα is a key regulator of lipid metabolism in liver, and it controls not only the gene expressions involved in mitochondrial β-oxidation but also those of downstream genes that affect FFA transport and synthesis in liver [36]. To further examine whether N800 treatment altered Pparα protein levels at three time points, we performed Western blotting. The protein levels of Pparα (Figure 11G) showed similar trends with the relative *Pparα* mRNA levels (Figure 10E, middle panel). There were persistent and significant decreases in Pparα protein expressions in the N800 group compared with the other groups at 48 h and 96 h (all *p* < 0.05). Thus, N800 treatment suppresses the expressions of transcription factors, including Foxa1, Srebp1c, and Pparα, and then induces hepatic MS, in line with the other types of drug-induced steatosis.

CYP2E1 is responsible for the metabolism of drugs and endogenous components in the liver. Studies have shown that NAFLD patients have increased expression of CYP2E1, and hepatic CYP2E1 is often induced in obesity and related metabolic disorders [37]. We analyzed whether high-dose NAC, which induced hepatic MS, affected the expression of Cyp2e1. In the N275 group, *Cyp2e1* mRNA at 24 h (Figure 11E) and Cyp2e1 protein (Figure 11F) levels at 24 h and 48 h were increased, but no significant difference was found compared with the C group. In the N800 group, *Cyp2e1* mRNA levels (24 h: *p* < 0.05; 48 h: *p* < 0.01) and Cyp2e1 protein levels (all *p* < 0.05) were persistently and significantly increased at all indicated time points when compared with the C group. Furthermore, Cyp2e1 protein maintained a high level at 96 h during hepatic MS formation (Figure 5, middle-center and -right panels). Therefore, we suggest that N800 treatment induces Cyp2e1 expression and then causes MS formation.

## 4. Discussion

**Figure 12:**
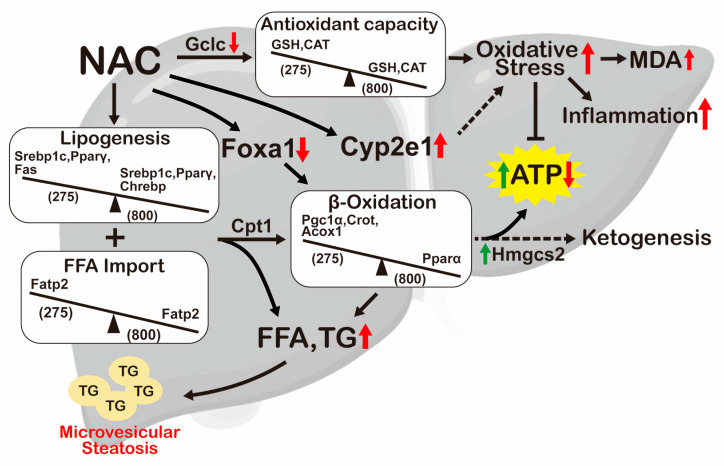
A molecular mechanism diagram of effects of a single IP of N275 and N800 on hepatic oxidative stress and lipid metabolism in normal mice. N275 treatment increases antioxidant capacity by elevating GSH level and CAT activity. In contrast, N800 treatment increases oxidative stress by decreasing GSH level (through inhibiting Gclc) and CAT activity, resulting in an elevated MDA level and inducing inflammation. In lipid metabolism, N275 treatment promotes de novo lipogenesis and FFA import through activations of Srebp1c, Pparγ, Fas, and Fatp2 expressions. N275 treatment also promotes β-oxidation by activating Cpt1, Pgc1a, Crot, and Acox1 expressions. Furthermore, the ketogenesis (through increasing Hmgcs2) and ATP production are increased in the N275 group (green arrows). However, N800 treatment inhibits de novo lipogenesis and FFA import by inhibiting Srebp1c, Chrebp, Pparγ, and Fatp2 expressions. N800 treatment also interferes with both mitochondrial (by inhibiting Pparα expression) and peroxisomal β-oxidations (by decreasing Crot expression), as well as decreasing ketone body synthesis (by decreasing Hmgcs2 expression), resulting in FFA and TG accumulations and low ATP production (red arrows). In addition, N800 treatment represses Foxa1 expression, which inhibits TG synthesis and accumulation and promotes peroxisome β-oxidation and ketogenesis, and thus promotes NAFL. Furthermore, N800 treatment directly or indirectly induces Cyp2e1 expression.

This study focused on the acute toxicity, causes of death, and potential toxic mechanisms of an IP of NAC in normal mice, especially the molecular mechanisms leading to hepatic MS (Figure 12). For an IP of NAC, the LD50 were 800 mg/kg for male and 933 mg/kg for female BALB/c mice. These LD50 results showed that male mice were more vulnerable than female mice to an IP of NAC. Since the LD50 of oral NAC of male mice is 6000 mg/kg [20], the safety dose of oral NAC is much higher than that of intraperitoneal NAC. An IP of male mice with the LD50 dose of NAC, N800, caused severe and potentially fatal damages to the liver, kidney, and spleen. Mice that survived the NAC overdose (≥800 mg/kg) showed chronic inflammation of the liver and kidney by serum biochemical indices. N800 treatment caused acute damages to the liver, kidneys, and spleen, indicated by histopathological analysis, which may explain the deaths of the mice. For the safe dose of NAC, N275, raised the hepatic GSH level but did not significantly alter the expressions of GSH synthetic enzymes. N275 treatment also improved CAT activity, lowered oxidative stress, and supported ATP synthesis. Furthermore, N275 treatment induced hepatic gene expressions involved in both lipid synthesis and β-oxidation, thus maintaining a balance between lipid catabolic and anabolic pathways. In contrast, regarding the LD50 for male mice, N800 treatment decreased the hepatic GSH level through suppressing expressions of GSH synthetic enzymes. N800 treatment also inhibited CAT activity. Thus, N800 treatment increased hepatic oxidative stress, impaired mitochondrial function, and then reduced ATP level. N800-treated mice were relatively weak, lost weight, and even died. Furthermore, N800 treatment lowered the hepatic gene expressions of lipogenesis and interfered with both the mitochondrial and peroxisomal β-oxidations, as well as decreased ketone body synthesis, leading to accumulations of TG and FFA, and then resulting in hepatic MS formation and Cyp2e1 induction. Based on the results of these studies, increased oxidative stress and impaired β-oxidation induce not only liver dysfunction, but also multiple organ failures due to mitochondrial inactivation.

Gender is an important factor leading to differences in pharmacokinetics and pharmacodynamics [38,39]. For example, many studies have shown that female mice are more resistant than male mice to APAP-induced hepatotoxicity [40,41,42,43]. The liver damage caused by excessive APAP is reduced in female mice [42], and female mice show a significantly higher LD50 of APAP than male mice [40]. Different factors have been identified as being responsible for the APAP resistance of female mice. The most likely factors include gene regulations of the quinone reductase, glutathione S-transferases Pi, and a gamma-glutamylcysteine synthetase heavy subunit by estrogen receptors [44]. Furthermore, the Gcl activity of female mice is higher than that of male mice, and then Gcl accelerates the recovery of APAP-depleted GSH levels and improves reactive oxygen detoxification [42,43]. Since male Gcl transgenic mice also exhibited resistance to APAP-induced liver injury when compared with wild-type male mice, these results indicate the critical roles of Gcl activity and gender in APAP-induced liver damage in mice [43,45]. These results also reinforce the importance of GSH in preventing APAP-induced hepatotoxicity. Otherwise, a pretreatment of 17β-estradiol in male mice can moderately decrease APAP-induced liver injury and oxidative stress by inhibiting neutrophil infiltration and stimulating the antioxidant defense system, without affecting GSH recovery [42,46].

Since high-dose NAC causes a similar GSH depletion as APAP overdose and then results in an increase in oxidative stress, we propose that female mice can quickly restore GSH levels because they have higher Gcl activity than male mice. The quick restoring GSH may be the primary mechanism by which female mice are more resistant to high doses of NAC. Furthermore, the high levels of 17β-estradiol, which stimulates the antioxidant defense system in female mice, may be the other possible mechanism. However, the actual mechanisms in female mice still need further analyses.

In previous studies, NAC labeled with isotopes was administered to rats or mice by IV or IP [47,48]. The NAC distribution profiles were similar between the IVs and IPs. NAC quickly and extensively accumulated in the liver, kidneys, and spleen, with the strongest NAC signal in the liver. The NAC signal peaked 45 min after an IV or IP in mice, and it was sustained for 5 h and still detectable at 24 h. This is in line with our results, that an IP of high-dose NAC caused mouse mortality mainly within 24–96 h post-injection, due to severe liver, kidney, and spleen injuries. Furthermore, an IP of N800 causes acute liver damage, as evidenced by the highest serum liver function markers, ALT/AST activities, at 24 h. As NAC was metabolized, plasma ALT/AST activities decreased significantly at 48–96 h, but the pathological score of liver damage increased over time, indicating that the liver damage is not transient. It is hypothesized that the hepatic NAC accumulation interferes with lipid metabolism and eventually leads to the induction of MS at 96 h. Previous studies have reported that MS induced by drugs (including NAC) results in liver failure and mortality in extreme cases [49]. Moreover, there were persistent NAC levels in the renal cortex and bone marrow at 24 h after an IP of NAC [48]. Based on the results of these works of literature and of this study, we propose that NAC accumulation in the renal cortex causes partial tubular necrosis and dilation, as evidenced by elevated serum kidney function indices and BUN/CREA levels. Furthermore, an NAC signal was present in the spleen at 24 h after IP [47,48]. We inferred that the blood volume stored in the spleen was decreased in the mice of the N800 group, causing spleen shrinkage. In summary, high-dose NAC is accumulated in the liver, kidneys, and spleen, so it causes liver, kidney, and spleen damages, leading to death or chronic liver and kidney inflammations in extreme cases.

Tissue damage is intensely influenced by oxidative stress, which arises from an imbalance between the production of ROS and the body’s capacity to neutralize or repair their detrimental effects. Prolonged oxidative stress leads to toxicity in various organs. Therefore, implementing strategies that manage oxidative stress and enhance antioxidant defenses are crucial for preventing and alleviating tissue damages and their subsequent health implications [50]. The main source of endogenous ROS was the electron transport chain, which can be neutralized with GSH and is mainly produced and exported by the liver, and antioxidative enzymes such as CAT; GSH and antioxidative enzymes play crucial roles in antioxidant defenses. GSH deficiency causes oxidative stress, which can lead to disease [51]. In hepatocyte-specific Gclc-knockout (KO) mice, GSH content in liver cells is decreased and cannot neutralize the ROS produced by mitochondria, leading to structural and functional changes in mitochondria, a reduction in ATP synthesis, hepatic steatosis, and lipid peroxidation. Finally, due to severe liver dysfunction, they die within one month [52]. In addition, in vitro experiments have demonstrated that the complete inhibition of GSH synthesis causes cell apoptosis [53]. NAC is a precursor for the synthesis of GSH. In this study, the safe dose of NAC (N275) indeed enhances hepatic GSH level and CAT activity, then resulting in an increase in ATP level in normal mice. However, the excess dose of NAC (N800) decreases hepatic GSH level through Gclc inhibition, and the histopathological effects of N800 treatment on the liver are similar to those seen in the previous Gclc-KO mice. Furthermore, hepatic steatosis was also detected in all GSH deficient mice, N800-treated mice, and Gclc-KO mice. In N800 mice, high levels of Cyp2e1 are maintained for at least 96 h, which is consistent with the high expressions of Cyp2e1 in both NASH patients and mice [37]. High levels of Cyp2e1 also metabolize fatty acids into cytotoxic lipid intermediates and then cause cell toxicity. Therefore, excess NAC is a pro-oxidant because it increases the oxidative stress. The molecular mechanisms of Gclc inhibition and Cyp2e1 induction by excess NAC require further investigations.

NAC ameliorates or prevents NAFLD by reducing oxidative stress and inflammation [2]. NAC also decreases hepatic lipid accumulation, elevates GSH level, enhances antioxidant capacity, and exerts therapeutic effects in NAFLD animal models [54]. However, in our previous publication, an NAC overdose induced an accumulation of hepatic lipids and the development of MS in both APAP-poisoning model mice and normal mice [22]. We compared the differences in the expressions of hepatic lipid metabolism-related genes between NAC-treated NAFLD mice in our previous study and normal mice in this study. In NAFLD model, the expressions of de novo lipogenesis genes (*Srebp1c* and *Pparγ*) and the FFA import gene (*CD36*) were induced by high-fat diet. NAC treatment inhibited high-fat diet-induced *Srebp1c* and *Pparγ* expressions, as well as *CD36* expression, and then decreased hepatic lipid accumulation [54,55]. In this study, N275 treatment induced, but N800 treatment inhibited, *Srebp1c* and *Pparγ* expressions in normal mice. Furthermore, in the mice fed with normal chow in our previous study, similar to the N275 group in this study, the lipid β-oxidation-related *Cpt1* and *Pgc1α* genes were induced by NAC [55]. Based on these comparative results, we hypothesize that NAC has different effects on hepatic lipid metabolism in normal mice and NAFLD model mice. Furthermore, N275 treatment promotes lipid metabolism and increases ATP level, while N800 treatment interferes with lipid metabolism, causing hepatic MS in normal mice. These results indicate that a single IP of high-dose NAC affects long-term lipid metabolism. Therefore, attention should be paid to the effects of different doses of NAC on hepatic and blood lipid metabolisms to prevent potential adverse reactions to an NAC injection. Moreover, in vitro studies have shown that NAC triggers mitochondrial-dependent apoptosis in cells [56]. Therefore, although NAC is highly beneficial for tissues and cells that require GSH replenishment, such as NAFLD liver cells, excess NAC impairs GSH synthesis in cells that already possess abundant GSH, such as the liver cells in normal mice.

The mitochondrial β-oxidation of fatty acids is essential for energy production and cell function. Some drugs, such as ibuprofen, tianeptine, amiodarone, tamoxifen, and valproic acid can impair this process by directly inhibiting specific enzymes and causing hepatic MS. This can lead to liver failure, renal failure, pancreatitis, brain dysfunction, coma, and even death [57]. The results of this study show that with an IP of N800 at 96 h, MS is developed mainly due to interference with Pparα, the transcription factor promoting mitochondrial β-oxidation. Moreover, the expressions of other downstream genes of Pparα, such as *Cpt1* and *Hmgcs2*, were also much lower in the N800 group than in the N275 group. In mice with liver cell-specific KO of Pparα, more severe hepatic MS is observed in fasting, aging, and NAFLD models [58], like the mice in the N800 group. The expression of PPARα, a target gene product for NAFLD treatment, is lower in NAFLD patients. Many natural extracts that may help with NAFLD are PPARα agonists [59]. Fibrate-activated PPARα promotes fatty acid mitochondrial β-oxidation and hampers the pro-inflammatory response in NAFLD [58]. PPARα agonists attenuate the IL-6-induced acute-phase response in vitro and in vivo [60]. Furthermore, IL-6 inhibits PPARα expression in human hepatocytes [61]. Since NAC blocks hepatic lipid accumulation in the animal models of NAFLD mainly through attenuating lipid peroxidation, enhancing intracellular antioxidant responses, and reducing pro-inflammatory markers, NAC is also a potential drug to treat NAFLD [2]. However, since few clinical studies support the beneficial effects of NAC on NAFLD-related complications, well-organized randomized clinical trials will be needed. In our previous study, N800 treatment induced IL-6 expression [22], but in this study it inhibited hepatic Pparα expression. Therefore, we suggest that N800 treatment induces IL-6, resulting in a reduced Pparα expression, and then interferes with mitochondrial β-oxidation, leading to MS formation in the liver.

Another PPAR family member, Pparγ, also influences lipid metabolism. Pparγ is not the main isoform expressed in the liver, and an overexpression of Pparγ in hepatocytes can lead to lipid accumulation, which may contribute to fatty liver [62]. Pparγ mainly increases hepatic FFA import (*CD36*) and lipogenesis (*FAS*)-related gene expressions. PPARγ expression also affects the inflammation occurred in NAFLD [63]. Increasing PPARγ expression can reduce or prevent the worsening of inflammation caused by NAFLD. In this study, N275 and N800 treatments showed opposite effects on both *Pparγ* and *Fas* expressions. N800 treatment intensely reduced *Pparγ* expression and increased inflammation, making liver metabolic syndrome worse over time and increasing pathological scores.

Various transcription factors associated with drug-induced steatosis share a common mechanism that involves the downregulation of Foxa1, Hex, and Srebp1c [35]. Foxa1 is a key regulator of hepatocyte lipid homeostasis, as it suppresses TG synthesis and enhances β-oxidation [64]. Moreover, FOXA1 expression has been found to be decreased in both human and rat NAFLD. Thus, Foxa1 is also a potential therapeutic target for NAFLD. Previous studies have also demonstrated that SREBP1c expression is inversely correlated with advanced NASH [65]. In this study, we observed that N800 treatment intensely reduces hepatic Foxa1 and *Srebp1c* expressions, leading to TG and FFA accumulations and resulting in hepatic MS. These findings are consistent with those of the above-mentioned studies. Therefore, N800 treatment may induce hepatic steatosis by impairing both Pparα-mediated mitochondrial β-oxidation and Foxa1/Srebp1c-mediated lipid metabolism gene expressions. However, the exact mechanisms and pathways by which N800 treatment modulates Foxa1, Srebp1c, and Pparα expressions and affects hepatic lipid accumulation remain to be elucidated.

## 5. Conclusions

The toxic effects of an IP of NAC on the liver, kidneys, and spleen are dose-dependent in normal mice. At excess doses (≥800 mg/kg), an IP of NAC causes organ dysfunction, fatty liver, renal tubular necrosis, splenic damage, and even death. The surviving mice show chronic inflammation of the liver and kidneys. At a safe dose (N275), an IP of NAC increases hepatic GSH level and CAT activity, resulting in elevated ATP levels. However, an IP of a high dose of NAC (N800) decreases GSH level and CAT activity, increases MDA level and oxidative stress, and then results in reduced ATP levels. N275 treatment upregulates the gene expressions related to FFA import, de novo lipogenesis, β-oxidation, and ketogenesis, while N800 treatment impairs these processes. N800 treatment decreases the expressions of genes that control FFA import, de novo lipogenesis and β-oxidation. As a result, N800 treatment causes severe hepatic accumulations of TG and FFA, and leads to hepatic MS.

## Figures and Tables

**Table 1:** Results of serum biochemical analyses of the control (C) male and female mice and the surviving (NAC) male and female mice after a single IP of high doses (≥800 mg/kg) of NAC for 14 days.

	C Male	NAC Male	C Female	NAC Female
Liver				
AST (U/L)	221.1 ± 40.5	185.7 ± 54.5	137.9 ± 42.2	262.3 ± 140.6
ALT (U/L)	88.0 ± 28.9	58.8 ± 29.3	36.9 ± 4.5	47.6 ± 17.1
ALP (U/L)	647.1 ± 25.8	441.8 ± 65.4 ***	435.6 ± 33.7	395.5 ± 44.3
T-BIL (μg/dL)	53.1 ± 15.9	26.7 ± 8.3 *	38.5 ± 2.6	13.9 ± 11.9 **
TP (g/dL)	4.7 ± 0.2	4.6 ± 0.2	4.8 ± 0.1	4.4 ± 0.1 ***
ALB (g/dL)	3.1 ± 0.1	2.7 ± 0.2 **	3.2 ± 0.1	3.0 ± 0.1 **
GLU (mg/dL)	186.5 ± 9.5	195.5 ± 26.9	181.8 ± 18.8	187.3 ± 35.4
Kidney				
BUN (mg/dL)	33.6 ± 4.6	27.1 ± 4.2 *	28.7 ± 1.6	26.8 ± 3.0
CREA (mg/dL)	0.14 ± 0.01	0.09 ± 0.03 *	0.13 ± 0.02	0.14 ± 0.03
UA (mg/dL)	1.9 ± 0.2	2.8 ± 0.5 *	1.9 ± 0.4	2.4 ± 0.6
Lipid				
TG (mg/dL)	113.7 ± 4.1	72.9 ± 10.0 **	79.5 ± 15.9	43.4 ± 8.3 ***
T-CHO (mg/dL)	176.6 ± 11.7	148.7 ± 10.5 **	125.2 ± 6.6	105.2 ± 6.9 ***
HDL (mg/dL)	131.4 ± 9.1	115.9 ± 9.2 *	94.7 ± 6.4	75.8 ± 5.4 ***
LDL (mg/dL)	29.6 ± 3.4	17.6 ± 3.9 ***	15.3 ± 2.5	14.6 ± 2.2
T-CHO/HDL	1.344 ± 0.089	1.283 ± 0.091	1.322 ± 0.070	1.388 ± 0.091
LDL/HDL	0.225 ± 0.026	0.152 ± 0.034 *	0.162 ± 0.026	0.193 ± 0.029
Ca Mg P				
Ca (mg/dL)	9.9 ± 0.2	9.5 ± 0.4	9.4 ± 0.2	8.6 ± 0.3 ***
Mg (mg/dL)	2.9 ± 0.3	2.7 ± 0.3	2.5 ± 0.1	2.7 ± 0.3
P (mg/dL)	9.2 ± 1.4	7.9 ± 1.5	5.9 ± 0.9	9.0 ± 1.5 **

Values of serum biochemical indices in the NAC male and female mice that show significant differences with the C male and female mice were labeled in bold. * (*p* < 0.05), ** (*p* < 0.01), and *** (*p* < 0.001) denote significant differences compared with the C group of the same gender.

## Data Availability

The data presented in this study are available on request from the corresponding author.

## Supplementary Materials

The following supporting information can be downloaded at: https://www.mdpi.com/article/10.3390/antiox13070832/s1. Table S1. Target transcript and primer sequences.
