# Supplementary material for: N-acetyl Cysteine Overdose Induced Acute Toxicity and Hepatic Microvesicular Steatosis by Disrupting GSH and Interfering Lipid Metabolisms in Normal Mice
Source: Antioxidants (Basel). 2024 Jul 11;13(7):832. doi: 10.3390/antiox13070832 (PMC11273582; doi:10.3390/antiox13070832)
Supplement: Supplementary file 1 [file antioxidants-13-00832-s001.zip › antioxidants-3070422-supplementary.pdf]

Table S1. Target transcript and primer sequences.

Target Transcript	Accession No.		Sequences
**18S rRNA**	NR_003278	Forward	5'- GTAACCCGTTGAACCCCATT -3'
		Reverse	5'- CCATCCAATCGGTAGTAGCG -3'
**Srebp1c**	NM_011480	Forward	5'- GCTGGCTTGGTGATGCTATGTTG -3'
		Reverse	5'- TATTACCTGGTGGAGGGCTGGAAGC-3'
**Chrebp**	NM_021455	Forward	5'- CTCAACGCTGCCATCAACTTG -3'
		Reverse	5'- GCTGAACACCCAGAACTTCCAG -3'
**Fas**	NM_007988	Forward	5'- CTTGGGTGCTGACTACAACC -3'
		Reverse	5'- GCCCTCCCGTACACTCACTC -3'
**Cpt1**	NM_013495	Forward	5'- AAGAACATCGTGAGTGGCGTCC -3'
		Reverse	5'- ACCCGAGAAGACCTTGACCATAGC -3'
**Pparα**	NM_001113418	Forward	5'- ATCAAGAAGACCGAGTCCGACG -3'
		Reverse	5'- GCAAATCCCTGCTCTCCTGTATG -3'
**Pgc1α**	NM_008904	Forward	5'- AGGGGCACATCTGTTCTTCCAC -3'
		Reverse	5'- CACTGGTCTTGTCTGATTTGTCGTC -3'
**Crot**	NM_023733	Forward	5'- CTGAGAGTGAAGGGCATTGTCC -3'
		Reverse	5'- ACTGGGTCCAACAGGTTCGTTC -3'
**Hmgcs2**	NM_008256	Forward	5'- CGTATGGGCTTCTGTTCAGTCC -3'
		Reverse	5'- TGTCAATGATGGTCTCGGTGC -3'
**Acox1**	NM_015729	Forward	5'- GCACCATTGCCATTCGATACA -3'
		Reverse	5'- CCACTGCTGTGAGAATAGCCGT -3'
**Foxa1**	NM_008259	Forward	5'- ATGGACCTCTTCCCCTATTACCGC -3'
		Reverse	5'- TGCCACCTTGACGAAACAATCG -3'
**Fatp2**	NM_011978	Forward	5'- ATCTGGCTGGGACTGCTCAAAC -3'
		Reverse	5'- CGTAAAAGACGGACACGGCATC -3'
**Pparγ**	NM_001127330	Forward	5'- GCCCTTTGGTGACTTTATGGA -3'
		Reverse	5'- GCAGCAAGGTGTCTTGGATG -3'
**Cyp2e1**	NM_021282	Forward	5'-AAGCGCTTCGGGCCAGG -3'
		Reverse	5'-TAGCCATGCAGGACCACGA -3'
